# Supplementary figures and images for: Evaluation of 68Ga-Radiolabeled Peptides for HER2 PET Imaging
Source: Diagnostics (Basel). 2022 Nov 5;12(11):2710. doi: 10.3390/diagnostics12112710 (PMC9689602; doi:10.3390/diagnostics12112710)

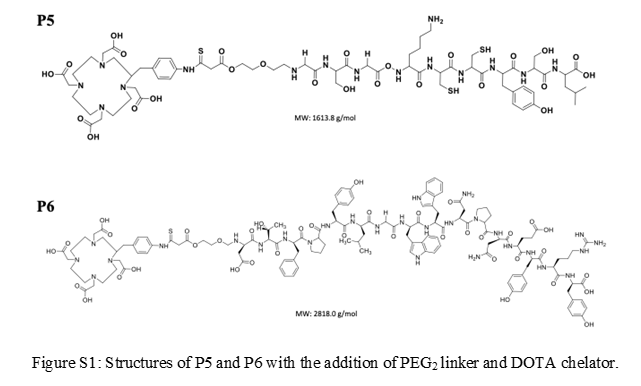

Supplement: Supplementary file 1 [file diagnostics-12-02710-s001.zip › Figure S1.TIF]

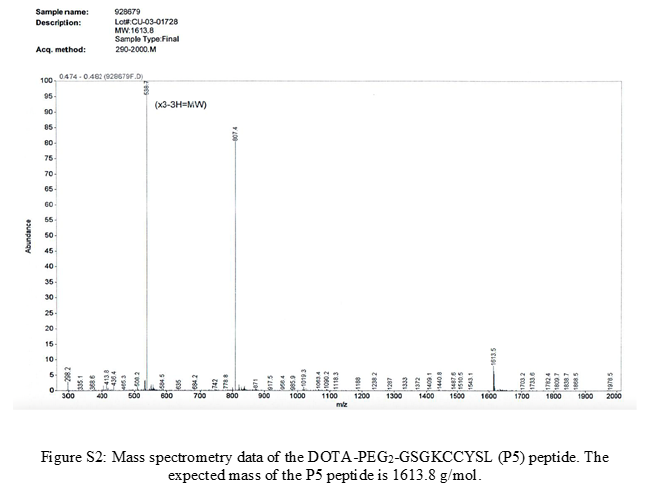

Supplement: Supplementary file 1 [file diagnostics-12-02710-s001.zip › Figure S2.TIF]

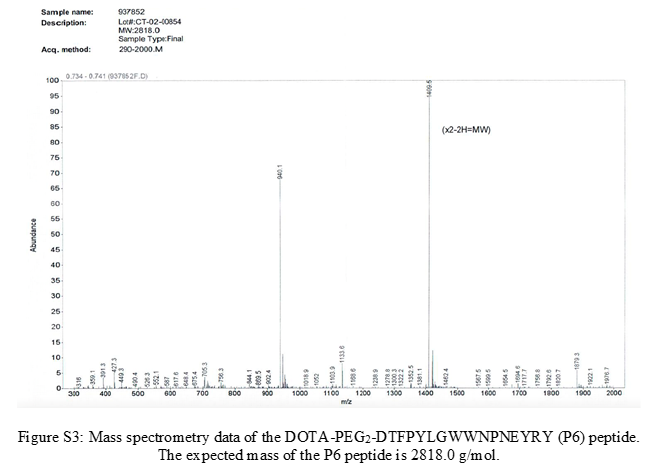

Supplement: Supplementary file 1 [file diagnostics-12-02710-s001.zip › Figure S3.TIF]

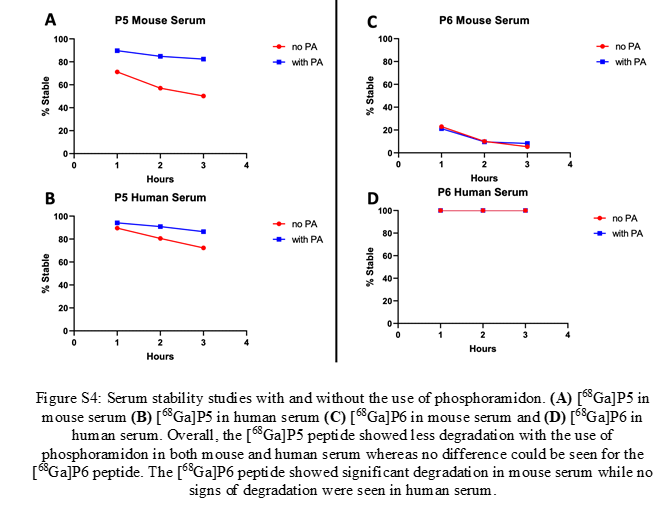

Supplement: Supplementary file 1 [file diagnostics-12-02710-s001.zip › Figure S4.TIF]

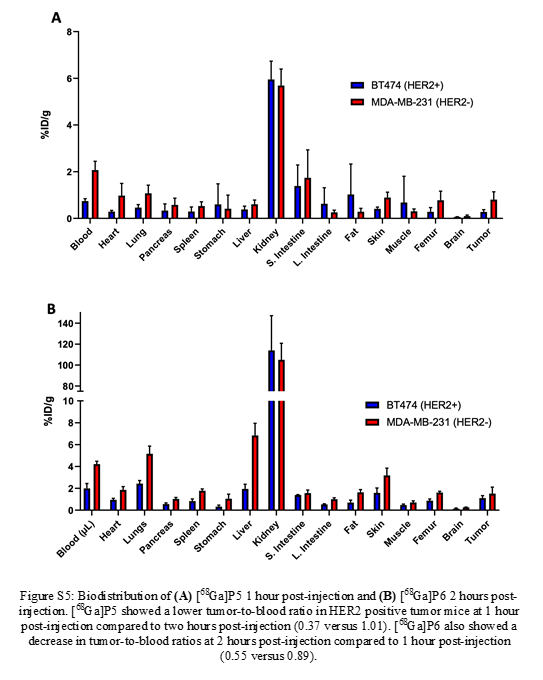

Supplement: Supplementary file 1 [file diagnostics-12-02710-s001.zip › Figure S5.TIF]

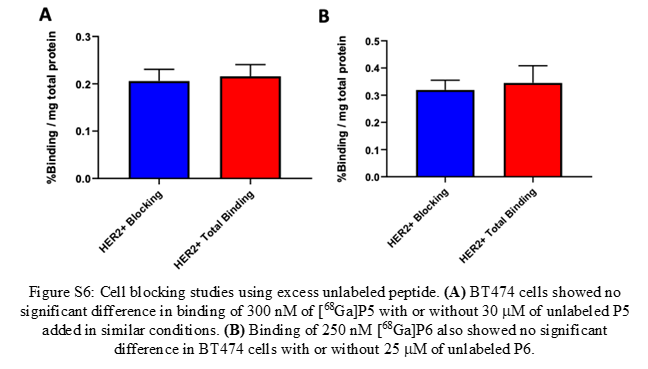

Supplement: Supplementary file 1 [file diagnostics-12-02710-s001.zip › Figure S6.TIF]

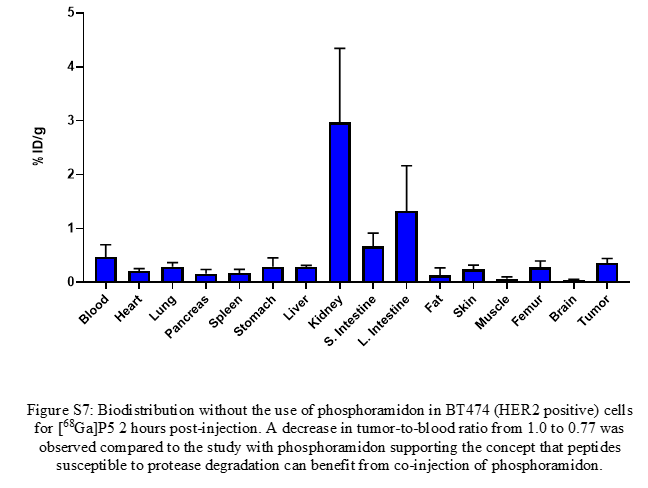

Supplement: Supplementary file 1 [file diagnostics-12-02710-s001.zip › Figure S7.TIF]

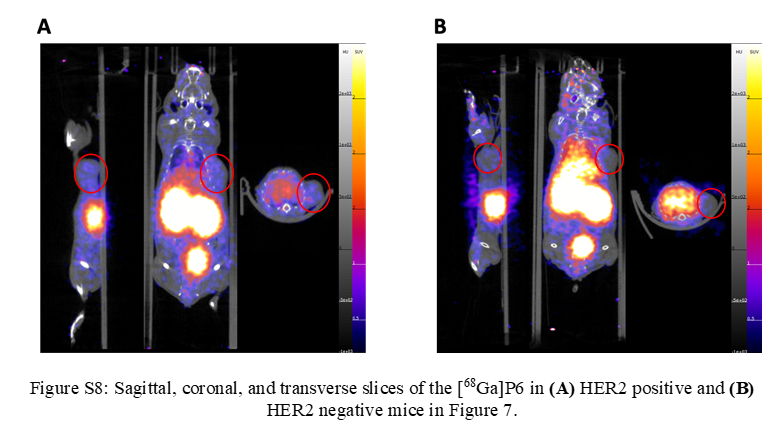

Supplement: Supplementary file 1 [file diagnostics-12-02710-s001.zip › Figure S8.TIF]
